# Closing the gap – detection of clinically relevant von Willebrand disease in emergency settings through an improved algorithm based on rotational Thromboelastometry

**DOI:** 10.1186/s12871-018-0672-8

**Published:** 2019-01-10

**Authors:** H.-G. Topf, E. R. Strasser, G. Breuer, W. Rascher, M. Rauh, F. B. Fahlbusch

**Affiliations:** 1 Department of Pediatrics and Adolescent Medicine, University of Erlangen-Nurnberg, Loschgestr. 15, 91054 Erlangen, Germany; 2 Department of Transfusion Medicine and Hemostasis, University of Erlangen-Nurnberg, Erlangen, Germany; 3 Department of Anesthesiology, University of Erlangen-Nurnberg, Erlangen, Germany

**Keywords:** Von Willebrand disease, VWD, Thromboelastometry, ROTEM, Emergency, Bleeding

## Abstract

**Background:**

Hemorrhage and blood loss are still among the main causes of preventable death. Global hemostatic assays are useful point-of-care test (POCT) devices to rapidly detect cumulative effects of plasma factors and platelets on coagulation. Thromboelastography (TEG) and Thromboelastometry (ROTEM) are established methods in many anesthesiological departments for guided hemostatic treatment. However, von Willebrand disease remains undetected by standard ROTEM, especially during emergency care, despite being the most prevalent congenital hemostatic disorder.

**Methods:**

In our monocentric cohort pilot study we focused on hemostatic challenges associated with von Willebrand disease. Twenty-seven patients with suspected von Willebrand disease were included. We modified the routine ROTEM assay by adding a preincubation with ristocetin and commercially available plasma-derived von Willebrand factor to identify clinically relevant von Willebrand disease (VWD).

**Results:**

Addition of von Willebrand factor to the ristocetin assay of a VWD type 3 patient restored the reaction of the whole blood probe to match the response of a healthy person. Our modified ROTEM assay with ristocetin (Ricotem) showed that all high responders (*n* = 7) had VWD. In the low responder group (*n* = 16) – 10 of 16 had VWD and in the normal responder group (*n* = 5), 2 of 5 had mild type 1 VWD.

**Conclusions:**

This new modification of the standard ROTEM assay enables the detection of otherwise unnoticed critical von Willebrand disease based on alterations in clot formation and might serve as a novel approach to reliably assess severe VWD patients by platelet-mediated blood clotting in an emergency setting. We recommend incorporating this new VWD-focused screening tool into the current ROTEM-based management algorithm of acute microvascular bleeding.

**Electronic supplementary material:**

The online version of this article (10.1186/s12871-018-0672-8) contains supplementary material, which is available to authorized users.

## Background

Hemorrhage and blood loss are still among the main causes of potentially preventable morbidity and mortality. The etiopathology of bleeding complications in trauma patients and other intensive care situations is diverse. Development of systematic hemostatic screening tests and of specific algorithms to guide hemostatic therapy for patients undergoing trauma surgery, emergency surgery and even elective surgery is ongoing [1–4].

Global screening assays for coagulopathy are regaining interest as point-of-care tests (POCT) based on their ability to mutually analyse the impact of plasmatic and cellular factors, such as platelets, white blood cells and erythrocytes. Thromboelastrometry (ROTEM) (ROTEM delta, TEM, Munich, Germany), Thromboelastography (TEG) (Haemonetics S.A., Signy, Switzerland), Sonoclot (Sienco Inc., Morrison, CO, USA) coagulation analysis [2, 5, 6], whole blood aggregation [7], whole blood thrombin generation [8] and clot waveform analysis testing [9] have the potential to dramatically change our current analytical approach to the assessment of hemostasis, due to their holistic emphasis on the physiology of coagulation. ROTEM and TEG were introduced to clinical practice as a POCT devices [10] and are both used as a diagnostic measure in various bleeding disorders in peri-operative clotting management [11, 12]. Thus, the viscoelastic approach became a broadly established method in many anesthesiological departments even beyond the operation room [13] and is widely used as it enables the temporal assessment of clot formation and dissolution. It has been modified to monitor antiplatelet medication. The effectiveness of ROTEM and TEG in diagnosing coagulopathy, as well as their clinical use for transfusion guidance and their predictive value for mortality in trauma patients has been thoroughly validated [14–16]. The use of ROTEM and TEG in the management of major bleeding has been described in recent guidelines [6].

Our work focuses on hemostatic challenges associated with the highly prevalent, mainly hereditary bleeding disorder von Willebrand disease (VWD). In contrast to its incidence that ranges from 0.1–1%, an estimate of only 1/50,000 and 1/8500 VWD patients requires specific treatment [17–21]. Von Willebrand factor (VWF) is an important glycoprotein involved in the hemostatic pathway and is organized in multimers. While acquired forms of VWD exist, these are far less common [22]. Different quantitative or qualitative deficiencies in VWF can cause various phenotypes of bleeding disorders all called VWD [23, 24]. The commonly used initial hemostasis tests, e.g. platelet count, activated partial thromboplastin time (aPTT) and prothrombin time (PT), have a low positive predictive value when screening for patients with an elevated hemorrhagic risk [3]. The diagnosis of VWD requires specific methods involving factor VIII, VWF antigen (VWF:Ag) and collagen binding (VWF:CB) levels. Classification and the definitive diagnosis of VWD, however, additionally depends on Ristocetin cofactor levels (VWF:RCo) and Ristocetin-induced platelet aggregation (RIPA), multimeric analysis of VWF and genetic analysis [17]. VWD is usually classified into three main types: Type 1 (quantitative deficiency), Type 2 (qualitative deficiency) which can be further classified into four subtypes (2A, 2B, 2 M, 2 N), and Type 3 (absence of VWF) [17, 24].

The use of TEG/ROTEM during surgery is well established [25–27], and age-related reference ranges for children [28] and neonates [29] have been evaluated. Von Willebrand multimers interact on many levels with endothelial cells, thrombocytes and fibrinogen. However, a whole blood ROTEM approach has not been used for closing the diagnostic gap for hemostatic alterations caused by VWD so far. TEG seems to have certain advantages over standard ROTEM in the detection of VWD [30]. In fact, standard ROTEM profiles of VWD do not differ from healthy controls [30]. Thus, our study describes a modification of the existing standard ROTEM assay to facilitate the detection of clinically relevant VWD in whole blood. Thereby, we aim at extending current emergency treatment options in an unclear bleeding situation.

## Methods

### Ethics approval

Ethical approval for this study (# 3763, as of 03/03/08) was provided by the local ethics committee of the University Hospital Erlangen, Erlangen, Germany. All participants signed an informed consent declaration. In cases where participants were legally minors (< 18 years of age), consent to participate was collected from the parents/guardians.

### Patients

Our prospective monocentric cohort study involved twenty-seven patients (*n* = 16 females, age range: 3 month - 61.7 years; *n* = 11 males; age range: 4 month - 38.8 years) who were referred to our medical centre for screening for hemostasis disorders. Testing was indicated by preceding bleeding episodes, findings of abnormal hemostasis tests prior to elective interventions or routine evaluation of previously diagnosed patients.

The participants were classified based on laboratory phenotyping [24]: i.e. VWF:Ag, VWF:CB and the analysis of multimers (all performed at the specialized coagulation laboratory of Prof. Budde, Hamburg, Germany). PT, aPTT and fibrinogen (Fib), Factor VIII, VWF:RCo and blood count were measured as a routine procedure at the Dept. of Adolescents and Pediatrics at the University of Erlangen-Nurnberg, Erlangen, Germany, using a XE-2100 analyser (Sysmex, Norderstedt, Germany) and the BCS XP System (Siemens Healthcare, Erlangen, Germany).

A threshold of 50% was applied to all laboratory tests concerning VWF to rule out VWD. If either VWF:Ag, VWF:CB, or VWF:RCo were below 50%, but other factors were above 50%, the potential presence of a mild type 1 VWD was considered. Thus, these patients were classified as possible VWD. Mild Type 1 was defined by VWF values between 30 and 50%, while the category of Type 1 is characterized by bleeding history or at least one VWF laboratory value below 30%. A VWF Ag below 3% was defined as Type 3, and Type 2 was defined by gel electrophoresis (performed by Budde Coagulation Laboratory, Hamburg, Germany, see below).

### Statistical analysis

Statistical analysis was performed using GraphPad Prism 7 (GraphPad Software Inc., La Jolla, CA, USA). For group comparison a two-tailed non-parametric Mann Whitney U test was employed. A *p*-value of < 0.05 was considered statistically significant. Results are given as median, 25th and 75th quartile and minimum/maximum value unless stated otherwise. Excel 2010, PowerPoint 2010 (Microsoft Corporation, Redmond, WA, USA) and Photoshop CS6 (Adobe Systems Inc., San Jose, CA, USA) were used for graphical layout.

### Laboratory testing

Viscoelastic measurements were performed as instructed with a ROTEM-Coagulation Analyser (ROTEM delta, TEM, Munich, Germany). Trisodium citrate was used as anticoagulant. Our modification protocol has been previously described in detail elsewhere [31].

In short, ROTEM measurements were performed with whole citrated blood. The remainder of the sample was centrifuged initially at 3000 g for 10 min. PT, aPTT, fibrinogen, FVIII:C (via an one stage assay) [32], VWF:Ag and VWF:RCo were determined by kit using commercially available reference plasma (all from Siemens Healthcare Diagnostics Products, Marburg, Germany). All measurements were carried out using the BCS XP System (Siemens Healthcare).

Gel electrophoresis, VWF:Ag and VWF:CB were performed at the reference laboratory (Budde Coagulation Laboratory, Hamburg, Germany).

### Thromboelastometry (ROTEM)

Rotational Thromboelastography (rTE), now known as rotational Thromboelastometry (ROTEM) [33], was performed at four channels simultaneously. Parameters included Clotting time (CT), Clot formation time (CFT), Maximum clot firmness (MCF) [34] and the Area Under Curve (AUC), as shown in Table 1. In short, after recalcification of citrated blood, different coagulation pathways were initialised using the commercially available agents Intem (contact pathway activation), Extem (tissue factor pathway) and Fibtem (fibrinogen dependent clot-formation via the tissue factor pathway, thrombocyte inhibition) (TEM, Munich, Germany) [31, 34].

**Table 1:**
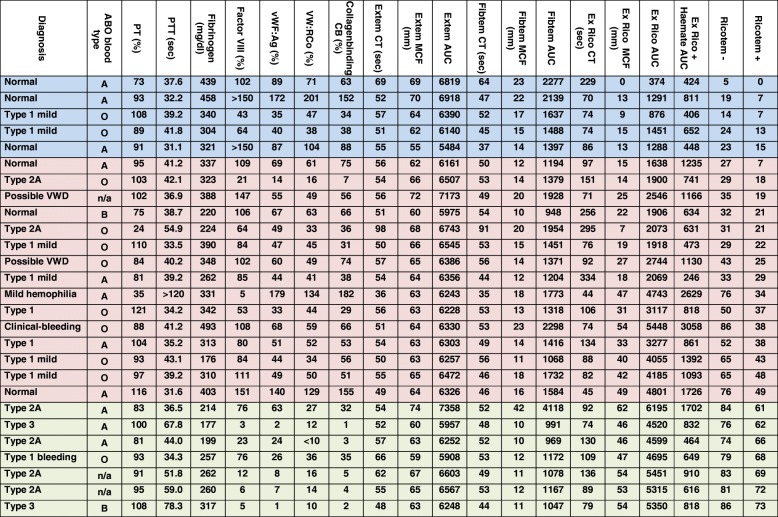
Overview of patient’s characteristics and hemostatic parameters

Color code: Blue = healthy subjects, red = low responders, green = high responders. Legend: ABO blood type: A, B, O and n/a as not available, sec = seconds. CT represents a measure of the initiation of clot formation, CFT represents the speed of clot formation, MCF is the value of the maximum clot strength and AUC is defined as the area under the velocity curve, i.e. the area under the 1st derivative curve ending at 30 min; PT (%) represents the prothrombin time, aPTT (sec) represents the activated partial thrombin time, VWF:Ag (%) represents the von Willebrand factor Antigen, VWF:RCo (%) represents the Ristocetin Cofactor activity, CB (%) represents the collagen binding activity of VWF, Extem represents the Extem activator reagent, Fibtem represents the Fibtem activator reagent, Ex Rico represents the assay, activated by Extem activator reagent after preincubation with ristocetin, Ex Rico + Haemate represents the assay, activated by Extem activator reagent after preincubation with ristocetin and Haemate® P, “Ricotem – ”is the calculation according to the formula: AUC_rico_/AUC_extem_ *100, “Ricotem +” is the calculation according to the formula: (AUC_rico_- AUC_rico + haemate_)/AUC_extem_ *100

The interquartile ranges for Ex Rico AUC were: 574 (25th perc.) -1463 (75th perc.); and for “Ricotem –”: 9.7 (25th perc.) -24 (75th perc.)

**Fig. 1:**
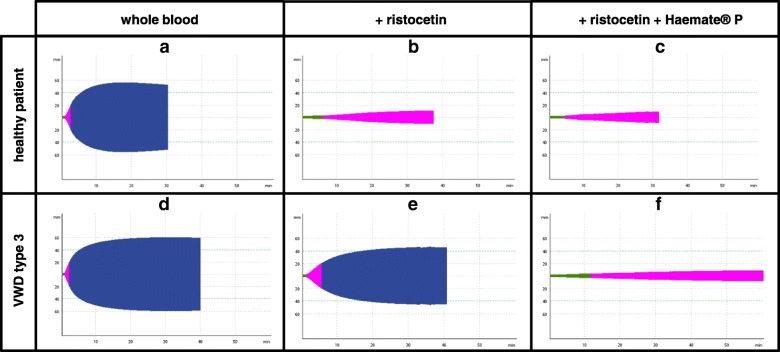
Representative graphical comparison of thromboelastometric (ROTEM, mm/min) results after consecutive addition of Haemate® P and ristocetin to whole blood in a healthy subject (**a-c**) and a patient suffering from VWD type 3 (**d-f**). Color code: Green = CT (clotting time), purple = CFT, blue = stable clot phase

a) Clotting after ristocetin platelet activation was analysed, as described in detail [31]:

In short, ristocetin (Trinity Biotech, Wicklow, Ireland) at a concentration of 15 g/L was added to the blood sample with subsequent incubation and mixing on a Spiramax 5 mixer (Ortho-Clinical Diagnostics, Neckargemuend, Germany). Afterwards we repeated the procedure as mentioned above. After recalcification the coagulation was started by the addition of Extem reagent. The resulting clot formation curve (AUC_rico_, Fig. 1 b, e) represents the clot strength, that remains after available platelets were reduced within the system via platelet agglutination through ristocetin prior to the standard EXTEM assay. The change in clot formation was calculated via the following formula: AUC_rico_/AUC_extem_ *100.

b) Clotting after ristocetin platelet activation and addition of VWF-containing concentrate:

Our novel modification of the established ROTEM method [31] outlined above, requires the addition of 5 μl of Haemate® P (CSL Behring) solution (0.8 IU) to 400 μl of each sample prior to the addition of ristocetin (see (a)). Afterwards procedure (a) was followed, as described above. The resulting clot formation curve (AUC_rico + haemate_, Fig. 1 c, f) represents the clot strength, which remains after available platelets were reduced within the system through platelet agglutination by ristocetin in the presence of additional plasma-derived VWF.

These assays resulted in three different AUCs, which we named after their modification: i) AUC_extem_ represents the regular Extem value; ii) AUC_rico_ represents the AUC values after addition of ristocetin; iii) for AUC_rico + haemate_ Haemate® P was added *prior* to the ristocetin assay. We used the AUC values 30 min after the ROTEM was started. The normal clot is formed by fibrin interacting with activated platelets. The mechanical strength and the viscoelasticity reflects the strength of this interaction [35]. The AUC values were used for comparison of the different modifications of the routine ROTEM procedure. To detect the effect of ristocetin on the whole blood coagulation process we calculated the ratio of the AUC_rico_ and the regular Extem (AUC_extem_).

Finally, for optimum comparison of results the difference of the respective AUC values was normalized to the corresponding Extem AUC values using the following formula: (AUC_rico_- AUC_rico + haemate_)/AUC_extem_ *100. If the addition of Haemate® P to the ristocetin assay has little effect, the formula returns values below 15%. In cases where AUC_rico_ equals AUC_rico + haemate_ the equation returns zero. However, if a substantial effect of Haemate® P is present the resulting values should increase up to 80%. 80% is expected to be the maximum value as approximately 20% of the clot strength is merely fibrinogen-dependent [35].

## Results

As shown in Table 1, twenty-seven patients were included in our study. Based on their laboratory values seven patients were identified as controls, nine were classified as VWD Type 1, including one patient with recurrent bleeding history, six were classified as Type 2A VWD and two patients were classified as Type 3 VWD. In two patients VWD was suspected (possible VWD, Table 1) based on the reduction of a single VW marker below its healthy reference value. One patient suffered from mild hemophilia (Table 1). For all patients the Extem, Fibtem, Extem with prior incubation of Ristocetin alone (i.e. Ricotem -) and the Extem with incubation of Ristocetin and Haemate® P (i.e. Ricotem +) were performed. The respective results are given in Table 1.

The straightforwardness of our novel ROTEM assay (especially of the graphical readout) is exemplary shown in Fig. 1, displaying representative ROTEM curves and AUCs retrieved from whole blood of a healthy subject (Fig. 1 a-c, *n* = 1) compared to a patient with diagnosed type 3 VWD (Fig. 1 d-f, *n* = 1). In the healthy subject (Fig. 1 a-c) addition of ristocetin resulted in a 94.5% reduction of the AUC (Fig. 1 b), when compared to baseline AUC_extem_ (100%, Fig. 1 a). Subsequent addition of Haemate® P to the assay increased the AUC_rico_ to 14.0% of the initial AUC_extem_ (Fig. 1 c). The addition of ristocetin to the representative patient with VWD type 3 (Fig. 1 d-f) on the contrary, only resulted in a minor reduction of the AUC by 24.1% (Fig. 1 e), when compared to baseline AUC_extem_ (100%, Fig. 1 d). Subsequent addition of Haemate® P to the assay, further reduced the AUC_rico_ to 6.2% of the initial AUC_extem_ (Fig. 1 f), similar to that of the representative healthy subject (Fig. 1c). Thus, addition of VWF (i.e. Haemate® P) to the ristocetin assay of a VWD type 3 patient was able to restore the reaction of the whole blood probe to match the response of the healthy control (Fig. 1 a-c).

**Fig. 2:**
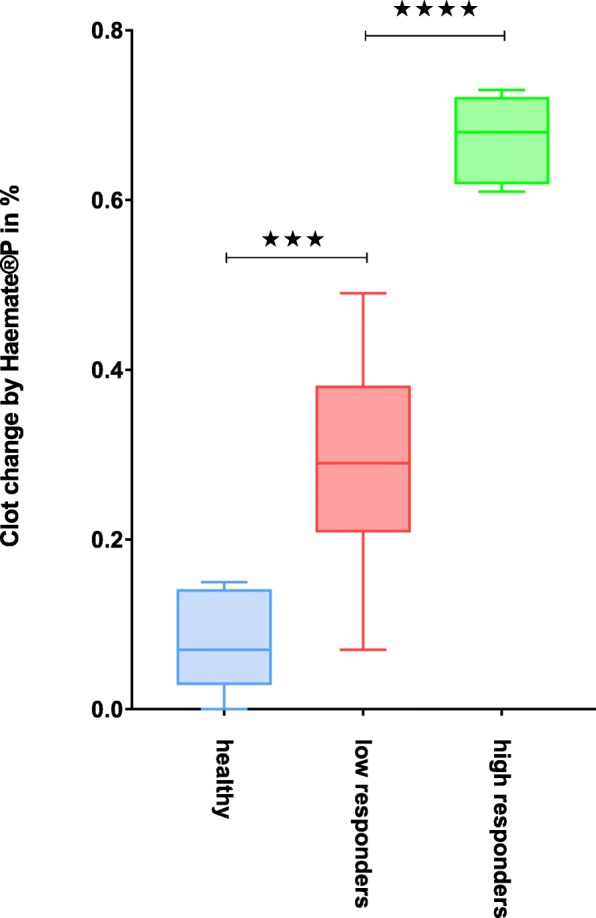
Statistical comparison of the response to Haemate® P in different groups. Legend: *** = *p* < 0.001, **** = *p* < 0.0001; Color code: Blue = healthy subjects, red = low responders, green = high responders

Next, we used a stepwise approach to assess the effectiveness our assay in all potential VWD-positive patients: Following the performance of AUC_extem_, AUC_rico_ and AUC_rico + haemate_ assays (Table 1), patients were grouped according to the result of their AUC_rico_/AUC_extem_ *100 (i.e. Ricotem -) values to discern healthy patients (Table 1, blue) from individuals with suspected VWD (Table 1, non-blue), according to the modified ROTEM, as published [31]. Subsequently, the two groups were subjected to further subgrouping according to their recovery rate (%) by Haemate® P addition, as calculated by the formula (AUC_rico_- AUC_rico + haemate_)/AUC_extem_ *100 (i.e. Ricotem +). The detailed results are displayed in Table 1. Healthy patients (as defined by Ricotem -) served as controls (Table 1, *n* = 5, blue). The remaining twenty-two patients with suspected VWD were further analysed with respect to their response to Haemate® P. While the reaction to the VWF was heterogeneous in *n* = 15 patients (Table 1, marked in red, so-called low responders), we found a clear-cut reaction in *n* = 7 patients (Table 1, marked in green, so-called high responders) to VWF. The statistical comparison of the clot change response to Haemate® P per group (Fig. 2) showed significant differences (*p* < 0.001 and *p* < 0.0001). As expected, some overlap between patients diagnosed as healthy and low responding subjects was visible, while the group of high responders (≥50%) could easily be distinguished (Fig. 2).

While further statistical analysis of potentially lower cut-off values was limited by our small cohort size we have supplied a graphical display of a lower cut-off value of 35% in Additional file 1: Figure S1. Additional file 1:Figure S1a represents all patients as color-coded depending on their group in Table 1 (cut-off 50%), which would qualify n = 7 patients for VWF treatment in an emergency setting at a specificity of 100%. In contrast, Additional file 1:Figure S1b displays the same patients, however the color-code was adjusted according to the lower cut-off value of 35%. This increased the number of qualifying patients by almost two-fold (*n* = 13) while reducing the specificity to 84% (11 of 13 patients).

## Discussion

Altered hemostasis and major blood loss largely contribute to potentially preventable morbidity and mortality in trauma patients or unexpected bleedings in the operation room [36], hence the term “hemostatic resuscitation” [37]. Current laboratory methods to diagnose severe coagulopathies are limited by laboratory runtimes. Viscoelastic analysis might solve many of these challenges, because it provides rapid information on clot stage and stability [13]. The efficacy of various hemostatic strategies has been intensively studied, as the massive transfusion of blood products might cause dangerous side effects. Thus, related guidelines mostly adhere to goal-directed strategies or algorithms [38–40]. Fibrinogen seems of high importance, but fibrinogen alone is not sufficient in all cases [41]. Early administration of hemostatic agents in clinical relevant bleeding, such as prothrombin complex concentrate (plasma- derived), fibrinogen (plasma derived or recombinant) or activated factor VIIa (recombinant), is proposed [42]. An increase of tranexamic acid and factor XIII usage was noted [43]. The administration of frozen plasma, DDAVP and thrombocytes are treatment possibilities for the bleeding patient. In our opinion the administration of VWF in emergency scenarios with bleeding patients has been neglected as a treatment option in those patients, so far. Correctly diagnosing VWD and in particular type 1 VWD, remains difficult [44]. The diagnosis of VWD is being further complicated by the fact that VWF is an acute phase protein [45] and thus a correct diagnosis can be masked in different clinical conditions. This circumstance was the incentive to further modify our initial laboratory-based assay to function as a bedside method with sufficient sensitivity and specificity to enable a reasonable implementation of VWF supplementation in an emergency setting [31]. Of course surplus transfusion of plasma and thrombocytes or sometimes DDAVP can be a good strategy in the treatment of a VWD-caused bleeding, but in some cases DDAVP could even harm the patient if VWD Type 2B is causative [46].

While the vast majority of VWD subtypes can be treated in a specialized manner by supplementing plasma-derived or recombinant VWF, for our proposed scope it is not necessary to diagnose the exact form of VWD at the emergency level. Of course, subtyping of VWD has to be done later in a specialized setting [47–50]. Nonetheless, a rapid diagnosis of a critical VWD, may it be inborn or acquired even, could be a great clinical advantage. Our novel ROTEM-assay might suggest a possible diagnostic cut-off for rapid identification of patients in need of VWF supplementation. As a limitation, however, our proof-of-principle study was performed on a limited number of pre-diagnosed patients in a non-emergency setting only.

Based on our previous study [31], we chose to utilize the clotting curve generated by the Extem activator reagent as reference to improve comparability to the work of others [51] and our own [31]. Nonetheless, the alternative or even additional use of Intem activator reagent seems feasible and could be taken into consideration, especially as Espinosa et al. could demonstrate that the correlation between conventional coagulation tests and ROTEM variables is subject to temporal change [52]. As shown by us earlier, the initial ratio (AUC_rico_/AUC_extem_ *100) already is of high prognostic value for the global function of VWF [31]. Further addition of the antibiotic ristocetin to whole blood or platelet rich plasma (PRP) initiates the binding of VWF to platelets, thereby generating platelet clumps. Subsequently, these newly formed platelet clumps are no longer available for the regular clotting reaction. This causes the AUC curve to decline in healthy patients, as fewer functional platelets are available. The effect of ristocetin on the clot is based on the presence of functional VWF. The clot strength of a blood sample, which was preincubated with ristocetin, is diminished compared to a blood sample without ristocetin. AUC_rico_/AUC_extem_ *100 describes the decrease of the AUC of the ristocetin inducible platelet agglutination. Thus, the effect of ristocetin and the likelihood of VWD prevalence are inversely related. Values of 100% indicate that ristocetin did not induce platelet agglutination. Based on our previous work [31] a cut-off value of 25% for Ricotem - was chosen. This resulted in an estimated sensitivity of about 65% and a specificity of 76% for the identification of VWD in our previous publication [31]. The greater the observed effect of additional VWF on the ROTEM AUC of a ristocetin-treated blood sample, the higher the likelihood that a bleeding patient could benefit from VWF treatment. For Ricotem +, we chose a cut-off value of 50% based on the graphical distribution of the recovery rate. However, in a clinical emergency setting, a lower cut-off (e.g. > 35%) might be eligible (Additional file 1: Figure S1a and 1b). Unfortunately, our study was limited by the low number of non-acute patients and our proof-of-principle approach only aimed to identify patients highly susceptible for major bleeding. Thus, future studies should involve larger cohorts to further enable statistical determination of Ricotem + cut-off ranges. Additionally, it remains to be determined, whether the quality of our test assay might benefit from the use of a recombinant VWF product instead of Haemate® P [50].

As monitoring of hemostasis has become increasingly crucial for acute bed side decision making [53], e.g. to improve the treatment of women with postpartum hemorrhage [54], the modification of established assays used either in the laboratory or as a POCT, can help to reliably diagnose coagulation problems. A modification of an established assay using factor XIII has already been published [51]. Small sample volumes might especially argue for the use of these viscoelastic devices in paediatric patients [28, 29]. As a limitation, ROTEM-based detection of platelet-dependent bleeding disorders can be significantly affected by numerous variables known to affect platelet function. These include, but are not limited to drugs or pathologic thrombocyte function [55]. This could explain why some of our patients with clinical hemorrhage showed increased AUC ratios, although we were not able to diagnose them as VWD later on. Hence further research is necessary for the clinical interpretation of results with values suspicious for VWD. Clearly, patients who were classified as “suspected VWD” by our assay would have to see a specialist for a safe diagnosis later. As VWF interacts with factor VIII in some cases, type 3 VWD could have been misdiagnosed earlier [56]. Prior to elective surgery all patients with suspected hemostatic disorders should visit a specialist for hemostasis allowing the diagnosis of severe VWD via the determination of VWF activity (GP1bM; GP1bR). In cases of acute bleeding that require fast clinical decision making without a confirmed diagnosis, however, the possibility of a loss of functional VWF should at least be taken into account.

**Fig. 3:**
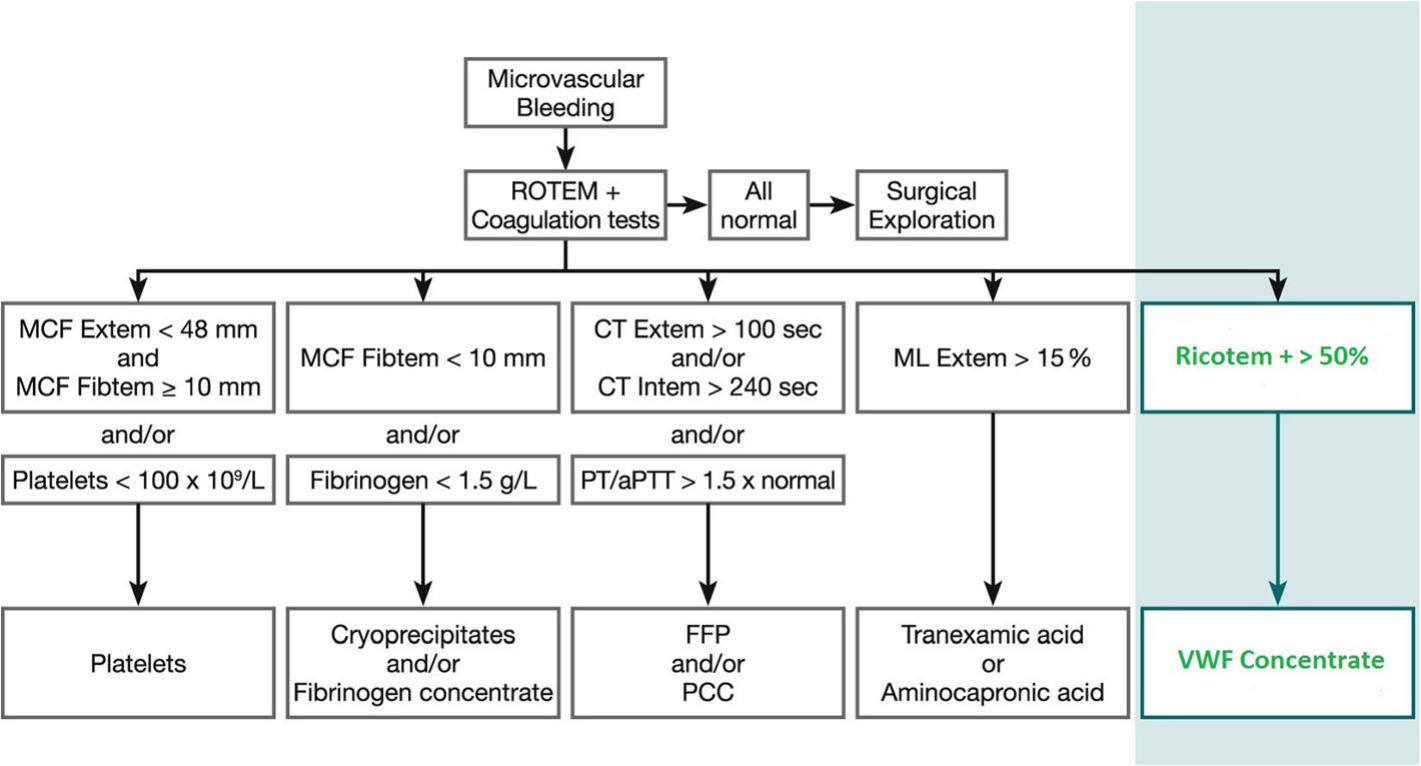
Modified management algorithm of microvascular bleeding from Bolliger et al. [2]: Based on the results of our presented ROTEM-technique we suggest an extension of the current version by an additional decision path (highlighted in blue)

For emergency settings Bollinger et al. elaborated a treatment algorithm based on routine laboratory results or ROTEM values [2]. We suggest adding another decision path (Fig. 3) to the proven flowchart [2] although further studies are needed to evaluate the benefits of this new thromboelastometric assay as a routine parameter. To stay in the tradition of the ROTEM names given to existing assays [34], we suggest the name “Ricotem +”. The Ricotem values represent the difference in clot stability through supplementing VWF within the ristocetin modified Extem assay. To proof a benefit of our new strategy a multicentre approach would be necessary. Additionally all patients with hemostatic disorders included in such a study should be genetically characterized.

## Conclusions

The main aim of our proof-of-principle study was to draw attention to VWF supplementation as a treatment option in trauma and emergency bleeding situations by providing ROTEM as an easy and helpful tool for deciding, if such supplementation could be reasonable. As plasma contains only low amounts of VWF, its substitution does not cover VWD sufficiently. The development of systematic hemostatic POCT tests is still ongoing, but to our knowledge, specific algorithms to guide hemostatic diagnostics and therapy for patients undergoing trauma/emergency surgery do not take the supplementation of VWF into account so far. VWD, the most prevalent congenital bleeding disorder, remains undetected by standard hemostatic testing and is difficult to detect in an emergency setting, if it has not already been diagnosed before. The addition of VWF (e.g. Haemate® P) to a well-established test assay provides a powerful tool to decide which patient should receive VWF in case of bleeding as an individualized treatment option. This extension of the current ROTEM-based bleeding management might help emergency physicians to recall this important, yet somewhat neglected treatment option.

## Additional file

Additional file 1: **Figure S1.** a) Patients response to Haemate® P. Values represent recovery in percent, as calculated via (AUC_rico_- AUC_rico + haemate_)/AUC_extem_ *100. Color code: Red = low responders, green = high responders. Cut-off 50% as in Table 1. Cut-off values (i.e. 50 and 35%) are indicated by solid black and orange horizontal lines, respectively. **Figure S1.**b) Patients response to Haemate® P. Values represent recovery in percent, as calculated via (AUC_rico_- AUC_rico + haemate_)/AUC_extem_ *100. Color code: Red = low responders, green = high responders with alternative cut-off value of 35%. Different cut-off values (i.e. 50 and 35%) are indicated by solid black and orange horizontal lines, respectively. (ZIP 83 kb)

## Abbreviations

aPPT: Activated partial prothrombin time
AUC: Area under the curve
CB: Collagen binding
CFT: Clot formation time
CT: Clotting time
MCF: Maximum clot firmness
Perc.: Percentile
POCT: Point of care test
PRP: Platelet rich plasma
PT: Prothrombin time
RIPA: Ristocetin-induced-platelet-aggregation
ROTEM: Rotational Thromboelastometry
Sec: Second(s)
VWD: Von Willebrand disease
VWF: Von Willebrand factor
VWF:Ag: Von Willebrand factor antigen
VWF:CB: Collagen binding assay
VWF:RCo: Ristocetin cofactor level
